# Three‐Dimensional Vascular Structure of Caudal and Dorsal Fins of a Dwarf Sperm Whale

**DOI:** 10.1002/ece3.70727

**Published:** 2024-12-16

**Authors:** Taro Okamura, Ashley R. Smith, Tomoyuki Mikami, Futaba Nishimura, Chika Shirakata, Kensuke Yasui, Shuichi Asakawa, Ken Yoda

**Affiliations:** ^1^ Graduate School of Environmental Studies Nagoya University Nagoya Japan; ^2^ Graduate School of Agricultural and Life Sciences The University of Tokyo Tokyo Japan; ^3^ Department of Geology and Paleontology National Museum of Nature and Science Tsukuba Japan; ^4^ Kanagawa Prefectural Museum of Natural History Odawara Japan; ^5^ Enoshima Aquarium Fujisawa Japan; ^6^ Laboratory of Veterinary Physiology Tokyo University of Agriculture and Technology Tokyo Japan; ^7^ Toyohashi Museum of Natural History Toyohashi Japan

**Keywords:** CT, dwarf sperm whale, fin, vascular structure

## Abstract

Previous studies have described two distinct vascular systems in cetacean fins. However, these studies have been limited to Delphinoidea species, with little information on their three‐dimensional structures. In this study, the anatomical analysis of the caudal and dorsal fins of a dwarf sperm whale was conducted using X‐ray computed tomography and gross dissection with staining, providing the first confirmation of the two vascular systems in the fins of the family Kogiidae. This finding suggested that these vascular systems are common across Odontoceti species. In addition, we observed unique three‐dimensional structures in the superficial veins, which formed a reticulate pattern in both fins and converged at the centerline at the base of the dorsal fin, potentially influencing individual's heat reduction capabilities.

## Graphical abstract

This study examined the vascular architecture in the caudal and dorsal fins of a dwarf sperm whale through X‐ray computed tomography and gross dissection. The analysis provided the first confirmation of two distinct vascular systems in the fins of the family Kogiidae, indicating their presence across Odontoceti species. In addition, unique three‐dimensional arrangements of superficial veins were observed, forming a reticulate pattern in both fins and converging at the base of the dorsal fin, potentially influencing thermoregulatory mechanisms.

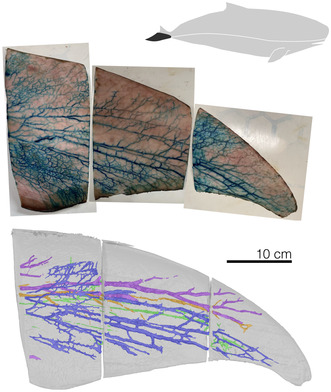

## 1 Introduction

Vascular systems in cetacean fins have attracted the interest of researchers as structures that contribute to thermal regulation in aquatic environments (Scholander and Schevill 1955; Elsner et al. 1974; Rommel et al. 1992; Rommel et al. 2006; Favilla, Adamczak, and Costa 2024). Cetaceans have two main types of vascular systems in the caudal and dorsal fins: periarterial venous retia (PAVR) and superficial veins (Scholander and Schevill 1955). PAVR consists of central arteries surrounded by veins connected directly to the aorta or vena cava, functioning as countercurrent heat exchangers that help maintain thermal stability in the fins. Superficial veins, on the other hand, serve as a “thermal window” for heat dissipation at the skin–water interface, potentially cooling reproductive organs by directing cooler blood through the lateral abdominal or caudal veins (Rommel et al. 1992, 1994; Rommel, Pabst, and McLellan 1993; Pabst et al. 1995). PAVR have been reported in many cetacean species, including Delphinoidea (Scholander and Schevill 1955), Eschrichtiidae (Elsner et al. 1974), and Balaenopteridae (Pabst, Rommel, and McLellan 1999). In contrast, superficial veins have only been reported in a few Delphinoidea species (Scholander and Schevill 1955; Rommel et al. 1992). Consequently, it is unclear whether both vascular systems are present in all cetacean families.

In addition, information on the three‐dimensional vascular structure within the fins is limited. Previous studies have relied mainly on two‐dimensional observations from gross dissection (Rommel et al. 1992) or X‐ray imaging (Elsner et al. 1974). PAVR in the dorsal fins of two Delphinidae species has been confirmed in three dimensions using magnetic resonance imaging (MRI), showing extensions toward the fin tips with a feathered branching pattern (Plön et al. 2018). In contrast, the observations of tiny vessels forming superficial veins remain incomplete, leaving their three‐dimensional structure unresolved. Textbooks on cetacean biology (Cozzi, Huggenberger, and Oelschläger 2016; Favilla, Adamczak, and Costa 2024) depict superficial vein branching in a divergent pattern at the superficial region of fins; however, these illustrations are based on planar observations, such as fin cross‐sections by Pabst et al. (1995) and Rommel et al. (1994), lacking direct evidence.

In this study, we document the three‐dimensional vascular structure of the caudal and dorsal fins of a fresh dwarf sperm whale *Kogia sima* using a combination of computed tomography (CT) and gross dissection with staining. The dwarf sperm whale, classified within the family Kogiidae, belongs to the most phylogenetically basal odontocete clade, Physeteroidea, which also includes the sperm whale *Physeter macrocephalus* (family Physeteridae) (McAlpine 2017; McGowen et al. 2020). Soft tissue morphological descriptions are lacking in Kogiidae due to its historical exclusion from whaling and the rarity of strandings, as its primary habitat is the deep sea (Bloodworth and Odell 2008). Huggenberger, Oelschläger, and Cozzi (2019) visualized a general vascular system in the body, excluding the region around the fins. However, to the best of our knowledge, the vascular system of Kogiidae fins has not been documented, even at the most basic level, such as the presence of blood vessels.

## 2 Materials and Methods

**FIGURE 1:**
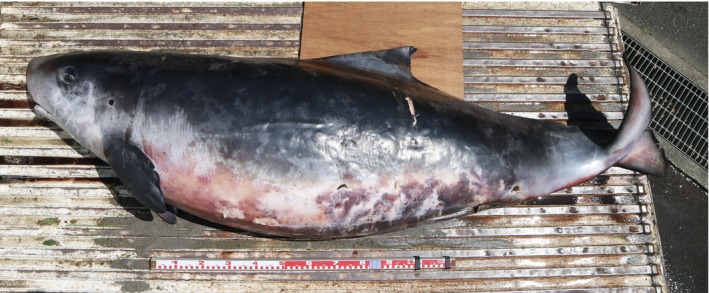
Lateral view of the external appearance of the dwarf sperm whale stranded on the coast facing Enshu‐nada, a part of the Pacific Ocean off the coast of central Japan.

A live‐stranded animal was found on the coast facing Enshu‐nada, Toyohashi, Aichi Prefecture, Japan (34°39′53.4″ N, 137°25′38.9″  E), on June 6, 2023, at approximately 8:00 a.m. The animal was pronounced dead shortly after discovery and was transported to the Toyohashi Museum of Natural History (TMNH). External measurement and dissection were performed on June 7, and the specimen was registered as TMNH‐MA‐668. The specimen, measuring 244 cm in body length, was identified as Kogiidae due to its small mandible relative to the maxilla and the distinctive “false gill” behind each eye (Figure 1, McAlpine 2017). Kogiidae includes pygmy sperm whales *Kogia breviceps* and the dwarf sperm whales. This specimen was classified as a dwarf sperm whale because its dorsal fin height was 8.6% of its body length (21.0 cm, Table 1), exceeding the 5% threshold (Caldwell and Caldwell 1989). A 429 bp sequence (Accession Number LC844731) was obtained through Sanger sequencing (Eurofins Genomics) and analysed using BLASTn‐matched LC741060.1, with 100% identity, confirming morphological identification. The specimen was indicated as a sexually mature female based on the presence of a uterus and its body length (Jefferson, Webber, and Pitman 2015). The caudal and dorsal fins showed no distinct wounds or healing marks and were frozen immediately after dissection.

**TABLE 1:** External measurements (cm) of the dwarf sperm whale specimen stranded on the coast facing Enshu‐nada, according to Norris and Prescott (1961); %BL = percentage of body length.

Measurement	cm	%BL
Body length	244.0	
Tip of upper jaw to angle of gape	12.0	4.9
Tip of upper jaw to blowhole	19.8	8.1
Tip of upper jaw to centre of eye	11.0	4.5
Tip of upper jaw to external auditory meatus	25.0	10.2
Tip of upper jaw to anterior insertion of flipper	34.0	13.9
Tip of upper jaw to tip of flipper	65.0	26.6
Tip of upper jaw to tip of dorsal fin	150.0	61.5
Tip of upper jaw to umbilicus	130.0	53.3
Tip of upper jaw to centre of genital aperture	163.0	66.8
Tip of upper jaw to centre of anus	174.0	71.3
Girth at axilla	152.0	62.3
Girth at anus	113.0	46.3
Length of flipper (Tip to anterior insertion)	34.5	14.1
Length of flipper (Tip to axilla)	25.3	10.4
Width of flipper	13.0	5.3
Dorsal fin length at base	50.0	20.5
Maximum height of dorsal fin	21.0	8.6
Maximum span of flukes	35.0	14.3
Width of fluke	21.0	8.6

**FIGURE 2:**
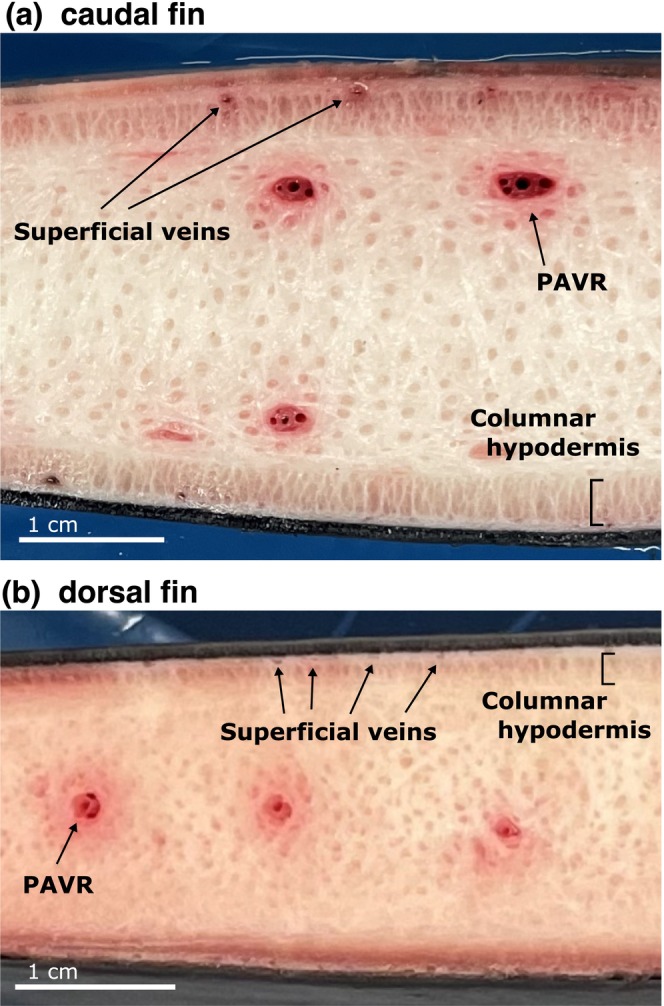
Approximate middle cross‐section of caudal and dorsal fins of the dwarf sperm whale. Two types of vascular systems with different structures, that is, periarterial venous retia (PAVR) and superficial veins, were located in the shallow and deep layers of columnar hypodermis, respectively. The position of the PAVR was different between the caudal and dorsal fins. See the *β* cross‐sectional images in Figure 3a (caudal fin) and 4a (dorsal fin) for full views of cross‐sections of both fins.

**FIGURE 3:**
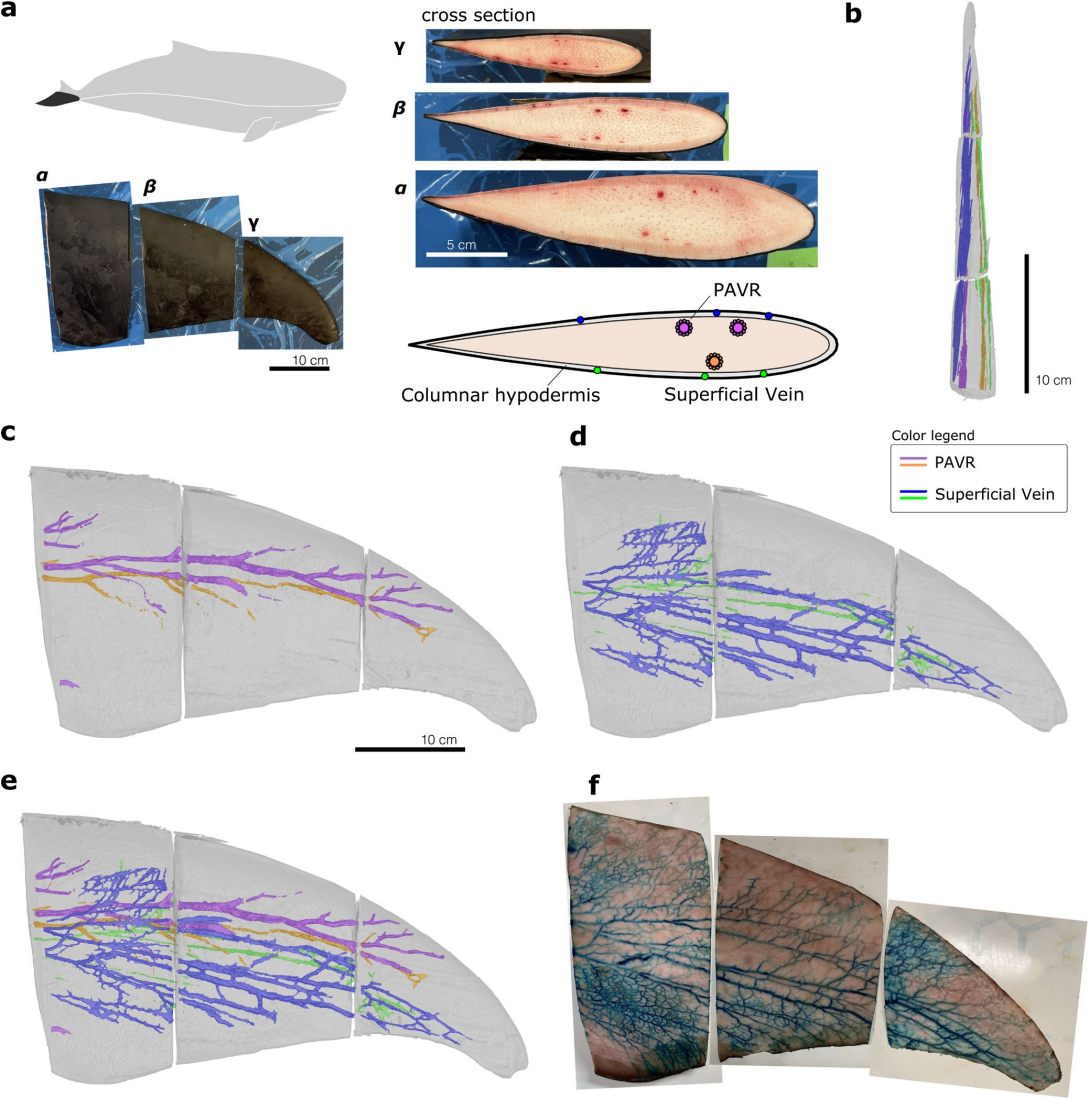
Results of the analysis of the vascular structure of the left caudal fin of the dwarf sperm whale. Dorsal view of external appearance, and images and schematic drawing of the cross‐section of the base (*α*) and cut surface (*β*, *γ*) of the caudal fin (a). Cranial (b) and dorsal (e) views of all vascular systems, dorsal views of PAVR (c) and superficial veins (d) using CT analysis of the caudal fin; dorsal view of stained and dissected superficial veins in the caudal fin (f).

**FIGURE 4:**
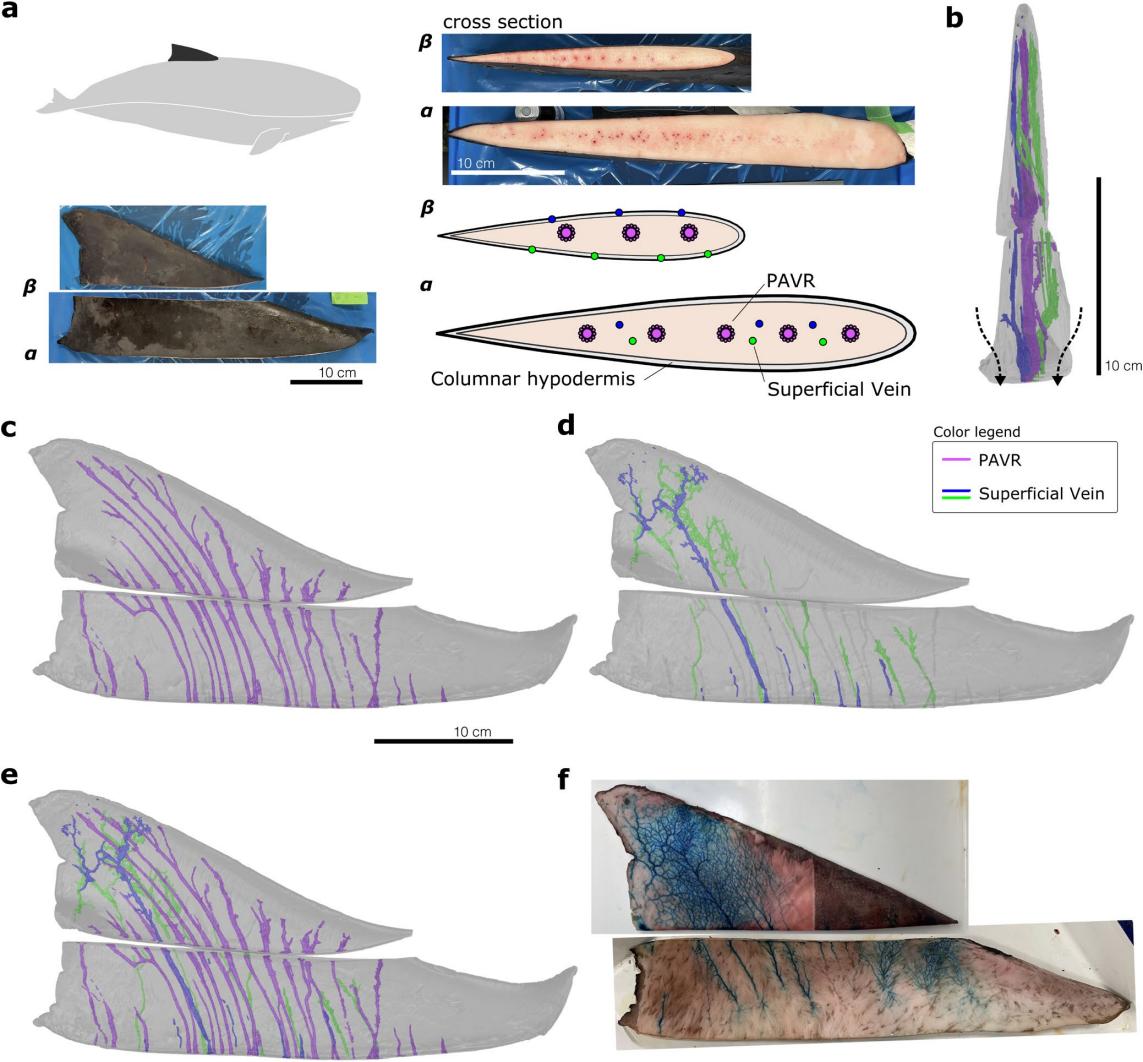
Results of the analysis of the vascular structure in the dorsal fin of the dwarf sperm whale. Lateral view of external appearance, and images and schematic drawing of the cross‐section of the base (*α*) and cut surface (*β*) of the dorsal fin (a). Cranial (b) and lateral (e) views of all vascular systems, lateral views of PAVR (c) and superficial veins (d) using CT analysis of the dorsal fin; lateral view of stained and dissected superficial veins in the dorsal fin (f). The arrows in (b) indicate the convergence of superficial veins.

The fins of the specimen were scanned using an X‐ray CT System (inspeXioSMX‐225CT FPD HR, Shimadzu Corporation) at the National Museum of Nature and Science, Tsukuba, Japan, on February 10, 2024. The fins were kept frozen until the day of scanning to prevent vessel rupture. The caudal and dorsal fins were divided into three and two sections, respectively, and the cross‐sectional structures of the fins and internal blood vessels were observed (Figure 2). The contrast agent was injected into all identifiable vessels on the cut surfaces, under high pressure to avoid incomplete penetration by valves or thrombi. Iohexol (Fuji Pharma Co. Ltd.) was diluted twofold and injected using 20G or 24G indwelling venous needles (Top Corporation). Tomographic images were acquired at 225 kV for the caudal fin, 199 kV for the dorsal fin, and 70 μA. The vessels were manually segmented from the CT images using Avizo (Thermo Fisher Scientific Inc.) and VGSTUDIO (Volume Graphics GmbH) (Figures 3b–e, 4b–e). Uniform staining was challenging because of thrombi, leading to visual‐based segmentation instead of threshold values, which affected the vessel thickness visualization. Rhino 8 (Robert McNeel & Associates Inc.) was used for visualization. In addition, vessels extending into the superficial layer of the fins were observed using manual dissection and staining. Methylene blue (Japan Pet Design Co. Ltd.) was injected directly into the vessels on the cut surface of the fins using a 27G needle (Top Corporation), following a method similar to that used in the CT analysis. After staining, the epidermis and dermal papilla were removed to expose the vessels in the superficial layer of the fins (Figures 3f and 4f).

## 3 Results

We obtained cross‐sectional images, three‐dimensional reconstructed models of blood vessels through X‐ray CT analysis, and images visualizing the vessels extending on the superficial layers of the fins with staining from fresh caudal and dorsal fins of the dwarf sperm whale. The results using each technique are described below.

### 3.1 Vascular Structure of Caudal Fin

The cross‐section revealed that the caudal fin mainly consisted of connective tissue, covered by a fibrous columnar hypodermis, dermis, and epidermis. Central arteries surrounded by veins were observed in the deeper layer of the columnar hypodermis, whereas individual vessels not surrounding arteries were observed in the shallower layers. These two vessel types with distinct cross‐sectional structures were symmetrically aligned along the anterior–posterior axis (Figures 2a, 3a). They were identified as the PAVR and superficial veins based on their structure and position. The vascular arrangement remained consistent from the base to the cut surface.

A three‐dimensional vascular reconstruction model of the caudal fin showed four vessels extending toward the tip (Figure 3b,e). The positions of these vessels in the model matched those in the cross‐section of the fin. The PAVR gently curves along the leading edge, branching in a feathered pattern from a pair of dorsoventral vessels at the fin base (Figure 3c). Superficial veins formed a reticulate pattern, repeatedly branching and connecting adjacent vessels and extending superficially toward the tip (Figure 3d). No apparent connections were observed between the PAVRs or between the PAVR and superficial veins, except near the tip, which was challenging to observe. Gross dissection with staining of the caudal fin showed that the vessels in the most superficial layer extended into the dermis (i.e., the shallower layer than the columnar hypodermis), forming a reticulate pattern (Figure 3f). These vessels were consistent with the superficial veins in the reconstructed model. However, the model could not fully capture all superficial veins, and stained dissection showed many more veins in a reticulate pattern extending complexly across the entire fin surface.

### 3.2 Vascular Structure of Dorsal Fin

The cross‐section of the dorsal fin was primarily composed of connective tissue, covered by the columnar hypodermis, dermis, and epidermis, similar to that of the caudal fin. Two types of vessels, identified as PAVR and superficial veins based on their cross‐sectional structure, were observed inside the dorsal fin but differed in position between the base and the middle cut surfaces (Figures 2b, 4a). No vessels were present in the shallower layers of the columnar hypodermis on the basal plane, where the PAVR and superficial veins were clustered at the centerline. In contrast, superficial veins lined the shallower layer of the columnar hypodermis on the cut plane, whereas the PAVR remained at the centerline.

A three‐dimensional vascular reconstruction model of the dorsal fin showed a single row of vessels at the centerline of the fin and symmetrical vessels in the shallow layers on the left and right sides (Figure 4b,e) positioned to match those in the dorsal fin cross‐section. More than 10 PAVRs at the centerline of the fin extended toward the tip, each gently curved, branching in a feathered pattern. Superficial veins were located at the centerline intermixed with the PAVR at the base of the fin. However, the veins shifted to the superficial region distally from 3.7 cm from the base (approximately 1/6 of height of the fin) and extended into the superficial region toward the fin tip, branching in a reticulate pattern (Figure 4b,e). No clear vessel connections were observed between the PAVRs or between the PAVR and superficial veins, except near the tip, which was challenging to observe. Gross dissection with staining of the dorsal fin revealed that the vessels in the dermis appeared at approximately 1/6 from the base (Figure 4f), and their arrangement was consistent with that of the superficial veins in the reconstructed model. However, the model could not fully capture all superficial veins. Stained dissection showed many more veins in a reticulate pattern extending complexly across the entire dorsal fin surface, similar to the caudal fin.

## 4 Discussion

Two vascular systems, the PAVR and superficial veins, were first observed in the caudal and dorsal fins of the dwarf sperm whale, consistent with those in Delphinoidea. The PAVR in the dorsal fin extended in a feathered pattern, similar to that of Delphinidae, as seen in MRI analysis (Plön et al. 2018). It can be said that the Kogiidae have a fin vascular system essentially identical to that of Delphinoidea, despite possessing ecological traits adapted to the deep sea. These findings suggest that the vascular system of the fins may reflect phylogeny more strongly than adaptation to their ecological niche and that the PAVR and superficial veins may be homologous across Odontoceti species, not limited to Delphinoidea. However, more detailed descriptions and comparative studies are required for many groups, including the Mysticetes, Physeteridae, and Ziphiidae, to fully understand the systematic evolution and adaptation of the fin vascular system.

The reticulate pattern in the superficial veins of the fins of the dwarf sperm whale was previously undocumented, differing from the radiating pattern shown in Rommel et al. (2006). However, we could not conclude whether this structure is species specific because of the absence of detailed descriptions of the three‐dimensional structure in previous studies. This did not appear to be an abnormality resulting from healing, as no damage or amputation marks were observed. Although not mentioned directly, a similar reticulate pattern was shown in figure 12A by Elsner et al. (1974), depicting the caudal fin of a bottlenose dolphin *Tursiops truncatus* . This suggests that reticulate structures may be more common across Delphinoidea species. The reticulate structure was only visible after staining as it was challenging to observe under normal conditions. The reexaminations including the Delphinoidea may be necessary to compare this structure across cetacean species using imaging techniques with contrast agents.

The superficial vein of the dorsal fin converged at the centerline of the base, in contrast to the superficial extension described by Rommel et al. (2006) (see figure 13 in the article). Similar to the reticulate structure, whether this structure is unique to dwarf sperm whales remains unclear. These findings suggested that the superficial veins penetrate the columnar hypodermis and that their direction is not influenced by it. Previous studies have shown inconsistent results regarding whether these veins extend shallowly (Elsner et al. 1974) or deeply (Scholander and Schevill 1955; Rommel et al. 1992) in relation to the hypodermis, which may be related to differences in observed fin cross‐sectional heights. More detailed cross‐species observations of superficial veins are needed. Dissecting these veins is challenging when they lie beneath the hypodermis, making three‐dimensional tomographic analysis valuable, as demonstrated in this study. However, tracking small reticulating vessels by using tomography remains challenging. Future research should combine CT techniques and gross dissection with staining to obtain a detailed description of the vascular structure.

Although functional comparisons between cetacean species are challenging, the structural characteristics of the superficial veins observed in this study likely influenced the heat‐releasing capacity of the fins of the dwarf sperm whales. Superficial vessels with branching in the reticulate pattern found in rabbit external ear (Clark and Clark 1934), toucan bill (Tattersall, Andrade, and Abe 2009), and *Stegosaurus* plates (Hayashi et al. 2012) are recognized as active thermal windows. The reticular pattern of these veins is thought to enhance heat release owing to their larger surface area compared to that of the divergent pattern. Functional differences between PAVR and superficial veins may be attributed to both cross‐sectional and three‐dimensional structures. In contrast, the convergence of superficial veins at the base of the dorsal fin may reduce heat dissipation efficiency if the vascular arrangement in the trunk is consistent with that of the Delphinoidea. Along with the deep‐sea adaptation, factors beyond heat release, such as structural function and embryological constraints, may need to be considered in understanding the formation of the three‐dimensional structure of the superficial veins. Further studies using varied observation techniques will clarify the thermodynamic function of superficial veins across and within species.

## Author Contributions

**Taro Okamura:** conceptualization (lead), formal analysis (equal), visualization (lead), writing – original draft (lead), writing – review and editing (equal). **Ashley R. Smith:** conceptualization (lead), formal analysis (lead), writing – original draft (equal), writing – review and editing (equal). **Tomoyuki Mikami:** formal analysis (lead), methodology (lead), software (lead), writing – original draft (equal). **Futaba Nishimura:** formal analysis (equal), writing – review and editing (equal). **Chika Shirakata:** formal analysis (equal), resources (lead), writing – review and editing (supporting). **Kensuke Yasui:** data curation (lead), investigation (lead), resources (lead), writing – review and editing (supporting). **Shuichi Asakawa:** funding acquisition (lead), project administration (lead), supervision (lead), writing – review and editing (supporting). **Ken Yoda:** funding acquisition (lead), project administration (lead), supervision (lead), writing – review and editing (supporting).

## Conflicts of Interest

The authors declare no conflicts of interest.

## Data Availability

The data supporting the findings of this study are available within the article and on MorphoSource: www.morphosource.org/projects/000669579; and Sketchfab: https://skfb.ly/p8SC6 and https://skfb.ly/p8SCp.
